# Expanding the Frontiers of Treatment: Cryoablation of an Ovarian Mass

**DOI:** 10.7759/cureus.12573

**Published:** 2021-01-08

**Authors:** Matthew Montanarella, Erik Soule, Carissa Concepcion, Travis Brown, Jerry Matteo

**Affiliations:** 1 Radiology, University of Florida College of Medicine, Jacksonville, USA

**Keywords:** ovarian tumor treatment, cryoablation, metastasis, minimally invasive, carcinoid tumor

## Abstract

The ovaries are a common site of metastasis from a variety of solid organ malignancies. These tumors most commonly originate from the gastrointestinal tract. Neuroendocrine tumors of the small bowel are unrelenting in their tendency to exhibit this type of distant spread, which poses a challenge for curative treatment. Whether metastatic disease to the ovary or primary ovarian malignancy, this is a major cause of morbidity and mortality for women of various ages. Currently, a mainstay of palliative treatment for advanced-stage disease resides in surgical debulking and chemotherapy. At times, these patients may not be surgical candidates due to various reasons which may include a large disease burden. Computed tomography-guided percutaneous cryoablation is a minimally invasive technique that has shown promise in treating solid organ metastatic lesions by exposing them to lethal temperatures. We describe a novel technique of palliative cryoablation of a primary small bowel carcinoid tumor that metastasized to the ovary. Hydrodissection was utilized to create a window for safe percutaneous treatment. At the end of freeze cycles, intraoperative CT was performed, demonstrating greater than 90% incorporation of the ovarian tumor within the margins of the lethal ice zone. Our team decided that this was a maximum percentage of freeze due to neighboring vessels and bowel. The patient tolerated this treatment well, and there were no reported post-operative complications. The procedure was clinically successful at shrinking the tumor as demonstrated on a nine-month follow-up CT. Percutaneous cryoablation is already a widely utilized method for treating tumors in various locations including the kidneys and liver. The application of cryoablation can be expanded as an effective and safe palliative technique for treating ovarian tumors. This may be especially useful in patients that are not surgical candidates.

## Introduction

Many women experience the uncertainty associated with the discovery of an ovarian mass. In patients undergoing a surgical procedure for removal of a mass, only 6-7% will find that their presumptive primary ovarian disease is a nongenital primary malignancy with metastasis to the ovary [1]. In the United States in 2018, 22,240 new cases of primary ovarian cancer were diagnosed [2]. In this same year, 14,070 women lost their lives to the disease [2]. The most common areas of metastatic disease to the ovary are gastric, breast, and colorectal cancers [3,4]. The prognosis of these patients is grim due to the advanced stage upon discovery of ovarian metastasis. Typical symptoms experienced by the patient often consist of abdominal pain, abdominal fullness, and dyspepsia. However, these patients may be entirely asymptomatic.

Carcinoid malignancy is among the etiologies that may exhibit this characteristic distant spread to the ovary. These neuroendocrine tumors arise from the enterochromaffin cells found within the gut. They metastasize through lymphatic channels and traditionally spread to the liver. Within the liver, the symptoms of carcinoid syndrome are exhibited by the excretion of serotonin [5]. These symptoms include diarrhea, flushing, and abdominal pain [6]. Traditionally, the treatment of distant metastatic disease from carcinoid and other primary nongenital tumors may include palliative chemotherapy and cytoreductive surgery. In a retrospective study of 154 patients with nongenital primary malignancy and ovarian metastasis, the overall median survival was 42 months [3]. In patients with optimal cytoreductive surgery through total abdominal hysterectomy with bilateral salpingo-oophorectomy, pelvic-paraaortic lymphadenectomy, and omentectomy, median five-year survival was 47% [3]. This is compared to those that received inadequate cytoreduction, in whom the median five-year survival of 23% was found [3]. Multifocal disease and large tumor burden may exclude the possibility of safe and adequate cytoreductive surgery in these patients.

Computed tomography-guided percutaneous cryoablation (PC) is a minimally invasive technique for treating soft tissue tumors. As the PC probe cools, crystals of ice form within the extracellular space within the surrounding tissue, which creates an area of hypertonicity that draws water out of cells. This action causes the lysis of cell membranes [7,8]. In areas that are near the periphery of the treatment zone, an apoptotic mechanism presides [8]. This therapy has shown to be comparable to traditional means of treatment in terms of outcomes and technical success. In a study of 590 patients with advanced-stage prostate cancer, those treated with percutaneous cryoablation had similar tumor control at seven-year follow-up compared to standard of care [9]. There are many advantages of cryoablation compared to traditional palliative treatment. PC allows for cytoreductive therapy in poor surgical candidates [10]. Reduction in tumor burden may improve prognosis in patients that have exhausted all other treatment options. PC has the potential to activate a robust tumor antigen-specific immune response following treatment [11]. An additional distinct advantage of cryoablation is allowing real-time monitoring of the ablation zone with intraoperative CT [12]. We describe the use of CT-guided percutaneous cryoablation on an inoperable biopsy-proven ovarian metastasis from an incompletely resected small bowel carcinoid. 

## Case presentation

**Figure 1:**
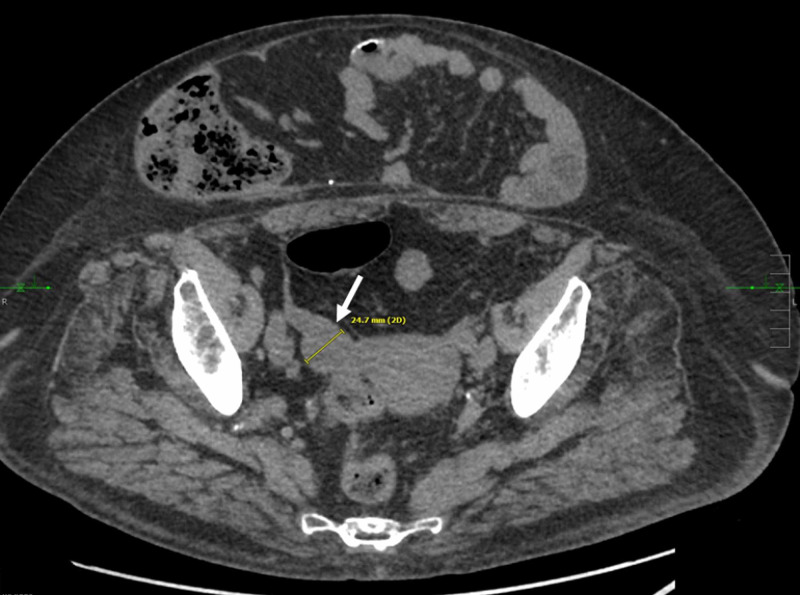
Routine surveillance CT demonstrating no evidence of metastatic disease of the right ovary Axial non-contrast CT obtained 36 months before ablation demonstrates normal right adnexal structures with a right ovary measuring approximately 2.5 cm.

The patient is a 67-year-old female that was diagnosed with a small bowel carcinoid tumor. Hepatic metastasis was detected after initial resection demonstrated incomplete margins. In the nine years since the discovery of distant metastasis, the patient underwent five PC hepatic cryoablations, five intra-arterial hepatic chemoembolizations, and trans-ventral hernia mesenteric cryoablation. Standard surveillance imaging throughout that time demonstrated a normal-sized ovary without evidence of metastasis (Figure 1).

**Figure 2:**
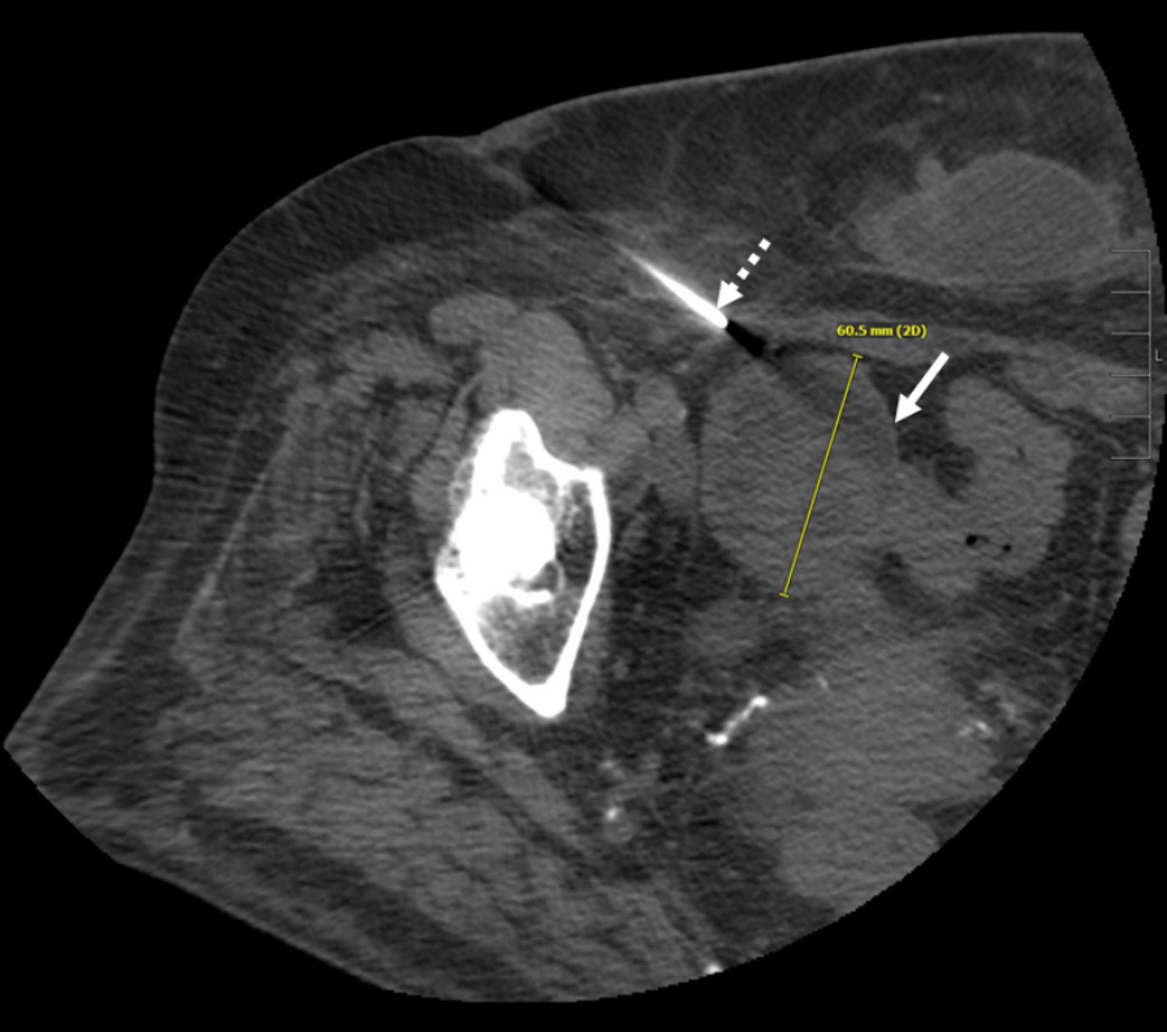
CT scan of the pelvis demonstrating percutaneous biopsy of the right ovarian mass Axial non-contrast CT obtained at time of biopsy six weeks prior to ablation demonstrates a well-circumscribed right adnexal mass (white arrow) measuring 6.0 cm in greatest dimension. Biopsy needle is seen from a right lateral approach (dashed arrow).

Subsequent follow-up cross-sectional surveillance imaging showed a mass on her ovary that measured 6.0 cm and biopsy confirmed carcinoid tumor metastasis (Figure 2).

**Figure 3:**
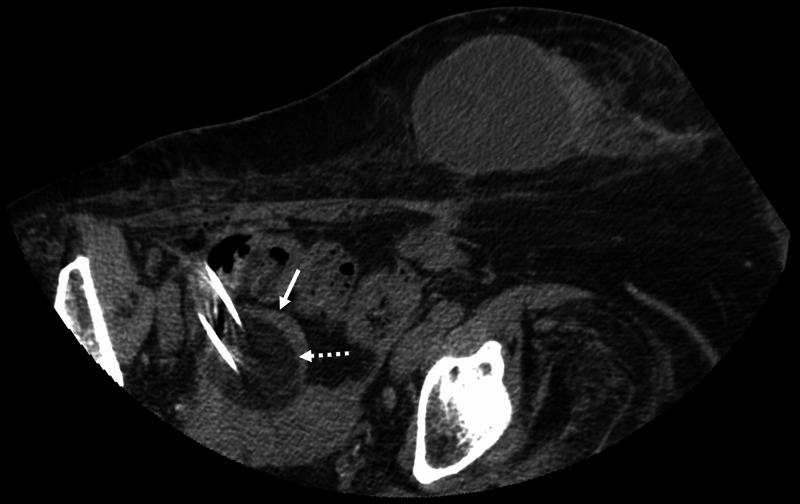
CT scan of the pelvis at the time of the cryoablation procedure Axial non-contrast CT obtained during cryoablation demonstrates positioning of two cryoprobes within the right adnexal mass (solid white arrow) along with formation of a hypodense “ice ball” encompassing the lesion (dashed white arrow).

The patient was positioned left lateral recumbent on the CT gantry and prepped and draped in the usual sterile fashion. Limited CT images were performed to delineate the right adnexal lesion for procedure planning. The skin was marked. Utilizing a 22-gauge spinal needle, saline was instilled around the lesion to provide hydro-dissection from adjacent colon and iliac vasculature. The cranial and caudal aspects of the mass were bracketed with two 17-gauge cryoablation probes inserted under CT guidance. Cryoablation was performed with a 10-minute freeze, followed by an eight-minute thaw, and followed by a 10-minute freeze. Imaging was obtained at five-minute intervals to evaluate the progression of the ice ball which ultimately demonstrated near-circumferential coverage of the right adnexal lesion (Figure 3).

**Figure 4:**
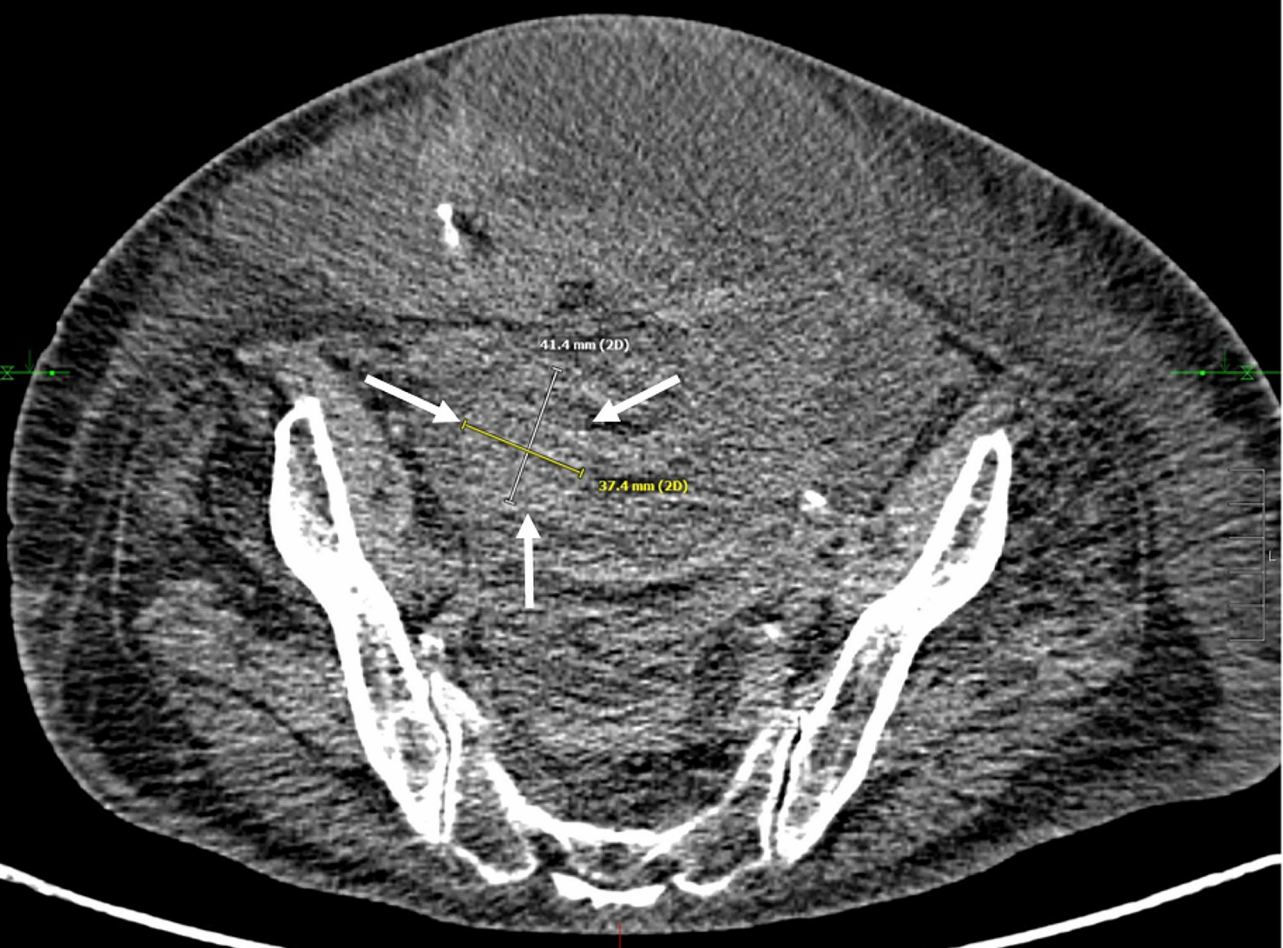
Surveillance CT nine months following cryoablation of the right ovarian mass Axial non-contrast CT obtained nine months post-ablation demonstrates decreased size of right adnexal mass (white arrows) measuring 4.1 cm in greatest dimension.

Following removal of the cryoablation probes, post-ablation CT revealed persistent hypodensity within the region of treatment without obvious injury to adjacent structures. The patient tolerated the procedure well and was discharged the same day after a brief period of post-procedure observation. Follow-up five-week CT demonstrated a decrease in the size of the ovarian lesion to 4.9 cm. The three-month CT showed a further reduction of the mass's size to 4.3 cm. At nine months, the mass decreased in size to 4.1 cm (Figure 4).

## Discussion

In patients with metastatic carcinoid tumors to the ovary that received oophorectomy, median survival was only 2.5 years with a five-year survival of 25% [13]. This is consistent with overall survival for metastatic neuroendocrine tumors with five-year survival between 19% and 38% [14]. Although carcinoid tumors primarily have a predilection towards spread to other organs such as the liver, this doesn't discount the utility of percutaneous cryoablation in debulking gynecological soft tissue metastasis or other primary and metastatic ovarian tumors. The use of systemic chemotherapy in patients with neuroendocrine tumors is not highly effective. Treatment response rates for single-agent and multi-agent regimens are 5-10% and 15-30%, respectively [15]. Surgical debulking remains a mainstay of treatment to maximize long term survival in patients with metastatic disease [13]. Due to improved diagnostic and treatment modalities, the survival of patients with metastatic carcinoid tumors has improved. This has led to uncharacteristic sites of metastatic involvement which may be amenable to treatment with PC [15]. 

Percutaneous cryoablation's analgesic effect due to freezing tissue and nerves makes for a less painful post-operative experience [5]. Cryoablation has demonstrated an ability to stimulate a potent inflammatory response in the treated tissue by releasing proinflammatory cytokines and immune cells. These immune cells and protein antigen framework left behind after treatment may potentiate long-lasting immune-mediated destruction of tumor cells in the ablation zone [7,16,17]. This immune response also can target and destroy tumor cells that are distant to the zone of ablation [7]. Finally, PC has an excellent safety profile due to the ability to monitor the ice ball intraoperatively. On rare occasions, cryoablation can lead to a systemic inflammatory response called cryoshock, a cytokine-mediated event that can cause cardiovascular compromise, multiorgan failure, and disseminated intravascular coagulation [7]. This adverse event is usually in response to a large area of liver ablation [7]. 

Few cases have been published on the use of cryoablation to treat primary malignancy of the esophagus, stomach, and colon. Rather, percutaneous cryoablation is most effective in the treatment of metastatic lesions of GI malignancy. Its efficacy, safety, and cost-effectiveness have been documented in increasing survival time in patients with metastatic colorectal cancers [18]. In a retrospective study of 30 patients with unresectable pelvic recurrent colorectal cancer, cryoablation was successfully completed in all patients with no serious post-operative complications [19]. The complete response rate in these patients was 77.14%, with a median duration of pain relief and progression-free survival of six months and ten months respectively [19]. Gastric cancer patients with liver metastasis had a median progression-free survival at six months of 59.2%, and these patients had a quality of life improvement following palliative cryoablation [20]. Although further studies will be needed to document response rates, we believe that the widespread use of percutaneous cryoablation can be effective in palliating ovarian metastatic GI malignancy.

The patient described in this article has been treated with multiple procedures over nine years to treat hepatic, mesenteric, and now ovarian metastases. She was deemed a poor surgical candidate because of the advanced tumor burden and a large ventral hernia. The utilization of percutaneous cryoablation has likely prolonged this patient's survival time. As far as these authors are aware, this is the first reported instance of PC being used to treat an ovarian neoplasm.

## Conclusions

The current gold standard for treating ovarian tumors is chemotherapy and open surgical debulking. As an alternative to traditional surgical debulking, CT-guided cryoablation served as a safe and effective means of treating nongenital metastatic ovarian malignancy in this patient.

To our knowledge, this technique has never been published in the literature. It is a viable debulking strategy for patients that are not surgical candidates. The preceding patient is currently alive and doing well 17 months after the procedure when traditional means of open surgical palliation were not offered. Future studies regarding the use of percutaneous cryoablation for the debulking of nongenital ovarian metastasis may prove it to be a proficient technique and new tool in palliative oncology.

## References

[1] Moore RG, Chung M, Granai CO, Gajewski W, Steinhoff MM (2004). Incidence of metastasis to the ovaries from nongenital tract primary tumors. Gynecol Oncol.

[2] Torre LA, Trabert B, DeSantis CE (2018). Ovarian cancer statistics, 2018. CA Cancer J Clin.

[3] Ayhan A, Guvenal T, Salman MC, Ozyuncu O, Sakinci M, Basaran M (2005). The role of cytoreductive surgery in nongenital cancers metastatic to the ovaries. Gynecol Oncol.

[4] Yamanishi Y, Koshiyama M, Ohnaka M (2011). Pathways of metastases from primary organs to the ovaries. Obstet Gynecol Int.

[5] Soule E, Bagherpour A, Matteo J (2017). Freezing Fort Knox: mesenteric carcinoid cryoablation. Gastrointest Tumors.

[6] Nandy N, Dasanu CA (2013). Management of advanced and/or metastatic carcinoid tumors: historical perspectives and emerging therapies. Expert Opin Pharmacother.

[7] Erinjeri JP, Clark TW (2010). Cryoablation: mechanism of action and devices. J Vasc Interv Radiol.

[8] Soule E, Matteo J (2018). Finally, a minimally invasive option for intrahepatic inferior vena cava invasion by hepatocellular carcinoma. Gastrointest Tumors.

[9] Bahn DK, Lee F, Badalament R, Kumar A, Greski J, Chernick M (2002). Targeted cryoablation of the prostate: 7-year outcomes in the primary treatment of prostate cancer. Urology.

[10] Littrup PJ, Bang HJ, Currier BP, Goodrich DJ, Aoun HD, Heilbrun LK, Adam BA (2013). Soft-tissue cryoablation in diffuse locations: feasibility and intermediate term outcomes. J Vasc Interv Radiol.

[11] Sabel MS (2009). Cryo-immunology: a review of the literature and proposed mechanisms for stimulatory versus suppressive immune responses. Cryobiology.

[12] Callstrom MR, Kurup AN (2009). Percutaneous ablation for bone and soft tissue metastases--why cryoablation?. Skeletal Radiol.

[13] Strosberg J, Nasir A, Cragun J, Gardner N, Kvols L (2007). Metastatic carcinoid tumor to the ovary: a clinicopathologic analysis of seventeen cases. Gynecol Oncol.

[14] Riihimaki M, Hemminki A, Sundquist K, Sundquist J, Hemminki K (2016). The epidemiology of metastases in neuroendocrine tumors. Int J Cancer.

[15] Zuetenhorst JM, Taal BG (2005). Metastatic carcinoid tumors: a clinical review. Oncologist.

[16] Derstine L, Soule E, Shabandi N, Arutyunova Z, Lall C, Scuderi C, Matteo J (2020). Rare treatment for a rare tumor: cryoablation of a granular cell tumor. Gastrointest Tumors.

[17] Ng KK, Lam CM, Poon RT (2004). Comparison of systemic responses of radiofrequency ablation, cryotherapy, and surgical resection in a porcine liver model. Ann Surg Oncol.

[18] Bang HJ, Littrup PJ, Currier BP, Goodrich DJ, Choi M, Heilbrun LK, Goodman AC (2012). Percutaneous cryoablation of metastatic lesions from colorectal cancer: efficacy and feasibility with survival and cost-effectiveness observations. ISRN Minim Invasive Surg.

[19] Wang Y, He XH, Xu LC (2019). CT-guided cryoablation for unresectable pelvic recurrent colorectal cancer: a retrospective study. Onco Targets Ther.

[20] Chang X, Wang Y, Yu HP, Zhang WH, Yang XL, Guo Z (2018). CT-guided percutaneous cryoablation for palliative therapy of gastric cancer liver metastases. Cryobiology.

